# *Mycobacterium avium paratuberculosis* Infection Suppresses Vitamin D Activation and Cathelicidin Production in Macrophages through Modulation of the TLR2-Dependent p38/MAPK-CYP27B1-VDR-CAMP Axis

**DOI:** 10.3390/nu16091358

**Published:** 2024-04-30

**Authors:** Muna M. Talafha, Ahmad Qasem, Saleh A. Naser

**Affiliations:** Division of Molecular Microbiology, Burnett School of Biomedical Sciences, College of Medicine, University of Central Florida, Orlando, FL 32816, USA; muna.talafha@ucf.edu (M.M.T.); ahmad.qasem@ucf.edu (A.Q.)

**Keywords:** vitamin D, CYP27B1, VDR, CAMP, Crohn’s disease, paratuberculosis

## Abstract

Background: Vitamin D plays a vital role in modulating both innate and adaptive immune systems. Therefore, vitamin D deficiency has been associated with higher levels of autoimmune response and increased susceptibility to infections. *CYP27B1* encodes a member of the cytochrome P450 superfamily of enzymes. It is instrumental in the conversion of circulating vitamin D (calcifediol) to active vitamin D (calcitriol). This is a crucial step for macrophages to express Cathelicidin Anti-microbial Peptide (*CAMP*), an anti-bacterial factor released during the immune response. Our recent study indicated that a Crohn’s disease (CD)-associated pathogen known as *Mycobacterium avium paratuberculosis* (MAP) decreases vitamin D activation in macrophages, thereby impeding cathelicidin production and MAP infection clearance. The mechanism by which MAP infection exerts these effects on the vitamin D metabolic axis remains elusive. Methods: We used two cell culture models of THP-1 macrophages and Caco-2 monolayers to establish the effects of MAP infection on the vitamin D metabolic axis. We also tested the effects of Calcifediol, Calcitriol, and SB203580 treatments on the relative expression of the vitamin D metabolic genes, oxidative stress biomarkers, and inflammatory cytokines profile. Results: In this study, we found that MAP infection interferes with vitamin D activation inside THP-1 macrophages by reducing levels of *CYP27B1* and vitamin D receptor (*VDR*) gene expression via interaction with the TLR2-dependent p38/MAPK pathway. MAP infection exerts its effects in a time-dependent manner, with the maximal inhibition observed at 24 h post-infection. We also demonstrated the necessity to have toll-like receptor 2 (TLR2) for MAP infection to influence *CYP27B1* and *CAMP* expression, as TLR2 gene knockdown resulted in an average increase of 7.78 ± 0.88 and 13.90 ± 3.5 folds in their expression, respectively. MAP infection also clearly decreased the levels of p38 phosphorylation and showed dependency on the p38/MAPK pathway to influence the expression of *CYP27B1*, *VDR*, and *CAMP* which was evident by the average fold increase of 1.93 ± 0.28, 1.86 ± 0.27, and 6.34 ± 0.51 in their expression, respectively, following p38 antagonism. Finally, we showed that calcitriol treatment and p38/MAPK blockade reduce cellular oxidative stress and inflammatory markers in Caco-2 monolayers following macrophage-mediated MAP infection. Conclusions: This study characterized the primary mechanism by which MAP infection leads to diminished levels of active vitamin D and cathelicidin in CD patients, which may explain the exacerbated vitamin D deficiency state in these cases.

## 1. Introduction

Vitamin D is a lipophilic hormone with several functions related primarily to calcium and phosphorus homeostasis that is achieved via an intricate interplay with the parathyroid hormone [1]. The major form of circulating vitamin D in the body is calcifediol, which undergoes hydroxylation via an alpha-hydroxylase enzyme known as cytochrome P450 family 27 subfamily B member 1 (*CYP27B1*) to synthesize the active form of vitamin D, calcitriol (1,25(OH)2D3) [2]. The latter can interact with its receptor, vitamin D receptor (*VDR*), to govern the expression of multiple genes, including the upregulation of cathelicidin antimicrobial peptide (*CAMP*) [3,4]. This cascade of interactions leads up to the synthesis of cathelicidin, and ultimately its active form LL-37, which has pleiotropic immune functions including anti-inflammatory and bactericidal effects that enhance the clearance of intracellular pathogens such as mycobacteria [4,5].

Crohn’s disease (CD) is an inflammatory bowel disease that leads to patchy foci of inflammation usually affecting the terminal ileum and colon portions of the gastrointestinal tract [6]. Worldwide clinical reports have established a high prevalence of this chronic condition in Europe and North America [7]. However, in the past few decades, incidence rates have also climbed in newly industrialized countries previously known to be low-risk populations [8]. The pattern of inflammation seen in CD affects all three layers of the gut epithelial lining; the mucosa, submucosa, and muscularis [9]. This full-thickness inflammatory infiltration results in a mechanical integrity compromise of the gut wall leading to fistulae formation, linear ulcers, and fissures [10,11]. Consequently, the inflamed and friable intestines become defenseless against several pathogens and immune triggers and lose many physiologic functions such as micronutrient absorption and maintenance of the gut microbiome [12,13]. Several CD manifestations such as malabsorption, pancolitis, and dysbiosis will then ensue, resulting in a notable malabsorptive derangement in CD patients, which involves fat-soluble vitamins like vitamins A, D, E, and K [14]. This occurs mainly due to a defective enterohepatic circulation of bile in the inflamed ileum [15].

*Mycobacterium avium* spp. *paratuberculosis* (MAP) seropositivity has been established in a subset of genetically susceptible CD cases [16,17,18]. Like other members of the mycobacteria genus, MAP possesses virulence factors that enable its survival inside the host cell leading to a state of overzealous inflammation [19]. Both MAP and *M. tuberculosis* (Mtb) infections were linked to dysregulation of the vitamin D metabolic pathway to avoid being detected by the immune system [4,20]. Mtb was found to rely on lipoprotein LprE to interact with TLR2 on the surface of macrophages to inhibit cathelicidin production through hypo-phosphorylation of the p38/MAPK pathway, which reduces *CYP27B1*, *VDR*, and *CAMP* expression [21]. This deploys several immunoevasive mechanisms such as preventing phagolysosome fusion, dysregulating autophagy, and T-cell function, in addition to modulating the secretion of various inflammatory mediators [21]. Our recent study indicated that LL-37 and calcitriol levels were reduced in MAP-positive serum samples of CD patients [14]. Moreover, LL-37 was found to play a role in mediating bacterial killing and inflammation clearance carried by calcitriol in MAP-infected macrophages [4].

The exact mechanism by which macrophages lose their ability to convert calcifediol to calcitriol remains uncertain. Thus, we were intrigued to investigate the effects of MAP infection on the TLR2-dependent p38/MAPK-CYP27B1-VDR axis and link those to the overall expression levels of *CAMP* in macrophages. The primary objective of this study was to propose a novel model in the treatment approach to MAP-positive CD patients that tackles the vitamin D metabolites, as well as the p38/MAPK inflammatory axis.

## 2. Materials and Methods

### 2.1. THP-1 Macrophages and Caco-2 Monolayers Cell Culture

THP-1 monocytes (ATCC TIB-202) were cultured in RPMI-1640 medium (ATCC 30-2001) supplemented with 10% fetal bovine serum (FBS; Sigma Life Science, St. Louis, MO, USA) and 0.05 mM 2-mercaptoethanol. Then, these cells were allowed to replenish and grow to confluency in cell culture flasks for 48 h at 37 °C in a humidified 5% CO_2_ incubator. A total of 5 × 10^5^ THP-1 monocytes per 2 mL media were plated in 12-well tissue culture plates and differentiated into tissue macrophages using 50 pg/mL phorbol 12-myristate 13-acetate (PMA; Sigma Life Science, St. Louis, MO, USA). We also used human enterocyte Caco-2 cells (ATCC HTB-37) that were grown in Eagle’s Minimum Essential Medium (EMEM) supplemented with 20% FBS (ATCC, Manassas, VA, USA) and maintained at 37 °C in a humidified 5% CO_2_ incubator for 7 days. When Caco-2 monolayers reached confluency, 5 × 10^5^ Cells per 2 mL media were plated in 12-well tissue culture plates or slides to allow differentiation for the next 14 days.

### 2.2. Infection and Treatment of THP-1 Macrophages and Caco-2 Monolayers

THP-1 monocytes were treated with either 50 ng/mL 25(OH)D_3_, or 50 ng/mL 1,25(OH)_2_D_3_ from Sigma Aldrich, St. Louis, MO, USA. These treatments were dosed twice directly into the culture media, once at the time of plating, and then at the same time as the MAP infection. Cells were also treated with three different concentrations (1 μM, 5 μM, and 10 μM) of SB203580 (MilliporeSigma, Rockville, MD, USA) at the time of plating. Cells were allowed to differentiate for 48 h following PMA treatment and the resulting THP-1 macrophages were infected with MAP UCF4 (1 × 10^7^ CFU/mL). The MAP UCF4 strain was originally isolated from an intestinal biopsy of a CD patient and was then cultured and maintained in a BD BACTEC Mycobacteria Growth Indicator Tube (MGIT 320) Automated System (Becton Dickinson and Company, Franklin Lakes, NJ, USA). Cells were harvested 24 h post-infection or treatment for RNA extraction. Supernatant from different THP-1 groups was used to indirectly treat or infect Caco-2 monolayers with MAP.

### 2.3. RNA Extraction, Reverse Transcription, and q-RT PCR to Measure CYP27B1, VDR, CAMP, NOX-1, IL-1β, and IL-10 in THP-1 Macrophages and Caco-2 Monolayers

Total RNA was extracted from THP-1 macrophages and Caco-2 monolayer cultures using an RNeasy Mini Kit (Qiagen, Hilden, Germany) at 24 h, and 48 h post-MAP infection. RNA was reverse transcribed to cDNA using a thermal cycler (MyGene Series Peltier). Gene expression was then measured using specific primers for *GAPDH* (housekeeping control gene to obtain baseline CT readings), *CYP27B1*, *VDR*, *CAMP*, *TNF-α*, *IL-1β*, and *IL-10* followed by a quantitative reverse transcription polymerase chain reaction (q-RT PCR). The primer sequences are confidential and proprietary information that can only be disclosed by Bio-Rad laboratories. Relative mRNA expression levels were calculated using the equation (2^(−∆∆CT)^).

### 2.4. Knockdown of TLR2 by siRNA Transfection

We prepared the knockdown transfection mix as described earlier by Vaccaro et al., 2022 [4], and Louis et al., 2022 [22]. Briefly, 5 nmol of Silencer™ pre-designed siRNA (siRNA ID: 4392420) specific to TLR2 was diluted in 50 μL nuclease-free water. An amount of 3.3 μL of this stock solution was diluted in 30 μL Optimem media (Gibco, Waltham, MA, USA). An amount of 9 μL of the resulting mixture was further diluted in 450 μL Optimem media. In a separate microcentrifuge tube, a 27 μL Lipofectamine reagent (Invitrogen, Carlsbad, CA, USA) was mixed with 450 μL Optimem media. This mixture was added to the 459 μL diluted siRNA mix. Each 2 mL of media in the 12 well-plate was treated with 300 μL of the transfection master mix. The knockdown was validated using a Human TLR2 ELISA kit (Ray Biotech, Norcross, GA, USA).

### 2.5. Measurement of Total and Phosphorylated p38/MAPK in THP-1 Macrophages

Total and phosphorylated p38/MAPK levels were measured 24 h after infection with MAP using a p38 MAPK (Total) Human ELISA Kit and a p38 MAPK (Phospho) [pT180/pY182] Multispecies InstantOne™ ELISA Kit (Invitrogen, Carlsbad, CA, USA). THP-1 macrophages were cultured and maintained as mentioned earlier and their lysates were collected and used to measure the total and phosphorylated p38/MAPK levels. The total p38/MAPK concentrations were measured in pg/mL according to the manufacturer’s protocol. The p38 MAPK (Phospho) levels were measured using the SkanIt Software Version 3.10.4 and plotted as absorbance readings multiplied by a factor of 10 at 450 nm reflecting protein abundance.

### 2.6. DHE Fluorescence Staining Assay for Caco-2 Monolayers

Caco-2 monolayers were grown at a density of 5 × 10^5^ cells/2 mL in Falcon 8-well chambered cell culture slides (ThermoFisher, Waltham, MA, USA) as described earlier [22]. DHE fluorescence staining was performed on the monolayers following 24 h of treatment with supernatant from MAP-infected THP-1 macrophages. Cells were first washed with PBS and then fixed with 10% formalin for 15 min. After fixation, the slides were washed with PBS twice and then treated with 1 μM DHE stain (Sigma Aldrich, St. Louis, MO, USA) for 25 min. The stain was removed, and the slides were washed with DI water twice and allowed to dry for 20 min. Finally, 4′,6-diamidino-2-phenylindole (DAPI; Vector Laboratories, Burlingame, CA, USA) was used to stain nuclei. Slides were examined under an Amscope IN480TC-FL-MF603 fluorescence microscope, where blue staining represents nuclei and red indicates oxidative stress. Images were captured on 100× magnification power and analysis was conducted by measuring the average integrated density using NIH Image J 1.39o software as described previously [22]. Merged images of DAPI and DHE stains were generated by the same software.

## 3. Statistical Analysis

Statistical analysis was performed on the data obtained from three biological (*n* = 3) and three technical (*n* = 3) replicates using GraphPad Prism V.7.02 software (GraphPad, La Jolla, CA, USA). Significance among experiments was assessed by a two-tailed nonparametric *t*-test. Results were expressed as mean ± SD unless otherwise mentioned. The significant difference between the experimental groups was referred to as *** *p* < 0.001, ** *p* < 0.01, and * *p* < 0.05. 

## 4. Results

### 4.1. MAP Infection Influences CYP27B1, VDR, and CAMP Expression in a Time-Dependent Manner and Calcifediol Reverses MAP-Induced Expression Trends

**Figure 1:**
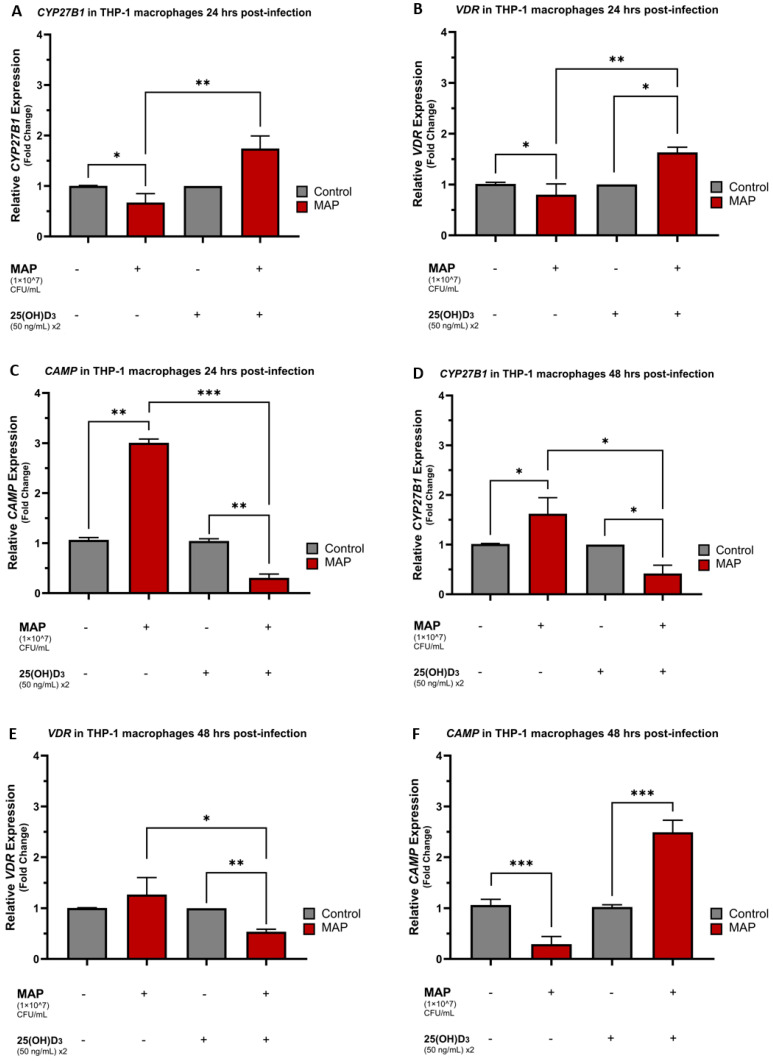
MAP infection and Calcifediol effects on *CYP27B1*, *VDR*, and *CAMP* expression in a time-dependent manner. Relative expression levels of *CYP27B1* in THP-1 macrophages 24 h (**A**) and 48 h (**D**) post-MAP infection. Relative expression levels of *VDR* in THP-1 macrophages 24 h (**B**) and 48 h (**E**) post-MAP infection. Relative expression levels of *CAMP* in THP-1 macrophages 24 h (**C**) and 48 h (**F**) post-MAP infection. 50 ng/mL 25(OH)D3 x2 indicates that THP-1 media was dosed twice with calcifediol (25(OH)D3), at plating and then at the time of the MAP infection. Relative expressions were quantified using RT-qPCR. Statistical analysis was performed on the data obtained from three biological (*n* = 3) and three technical (*n* = 3) replicates. * Indicates *p*-value of less than 0.05. ** Indicates *p*-value of less than 0.01. *** Indicates *p*-value of less than 0.001.

THP-1 macrophages infected with MAP for 24 h had lower relative expression levels of *CYP27B1* and *VDR* compared to the control, non-MAP infected macrophages with a statistically significant average fold change decrease of 0.67 ± 0.18 and 0.80 ± 0.21 (*p*-value < 0.05), respectively (Figure 1A,B). MAP infection also resulted in an increased expression of *CAMP* 24 h post-infection relative to the control group with a mean fold change increase of 3.00 ± 0.08 (*p*-value < 0.001) (Figure 1C). Treatment with calcifediol resulted in the reversal of these gene expression trends with an upregulation of *CYP27B1* and *VDR* expression and downregulation of *CAMP* expression 24 h post-infection. At the 48 h post-infection time point, there was an increased relative expression of *CYP27B1* and *VDR* (1.62 ± 0.32 and 1.27 ± 0.34, respectively) (Figure 1D,E) and a decreased relative expression of *CAMP* (0.29 ± 0.15) (Figure 1F). Calcifediol treatment also reversed MAP effects at 48 h post-infection, with a decreased expression of both *CYP27B1* and *VDR* and an increased relative expression of *CAMP*.

### 4.2. TLR2 Is Necessary for MAP Infection to Hinder Vitamin D Activation and Cathelicidin Production

**Figure 2:**
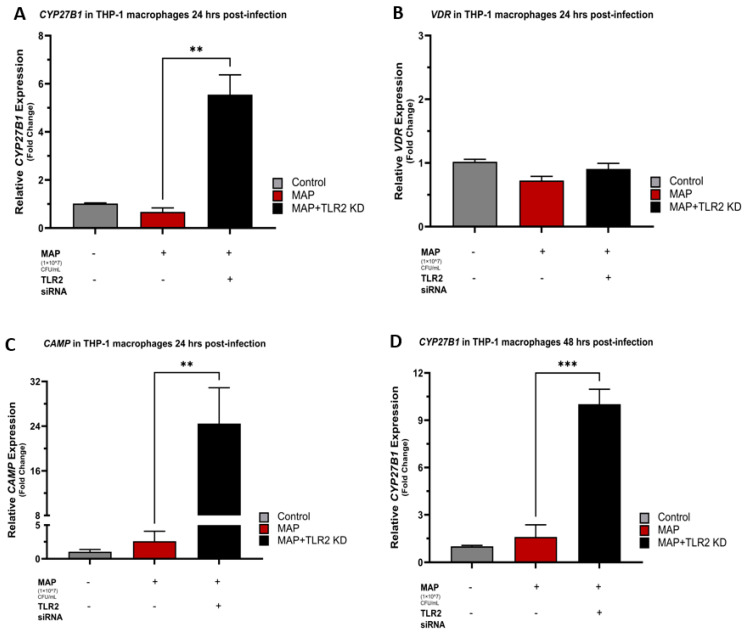

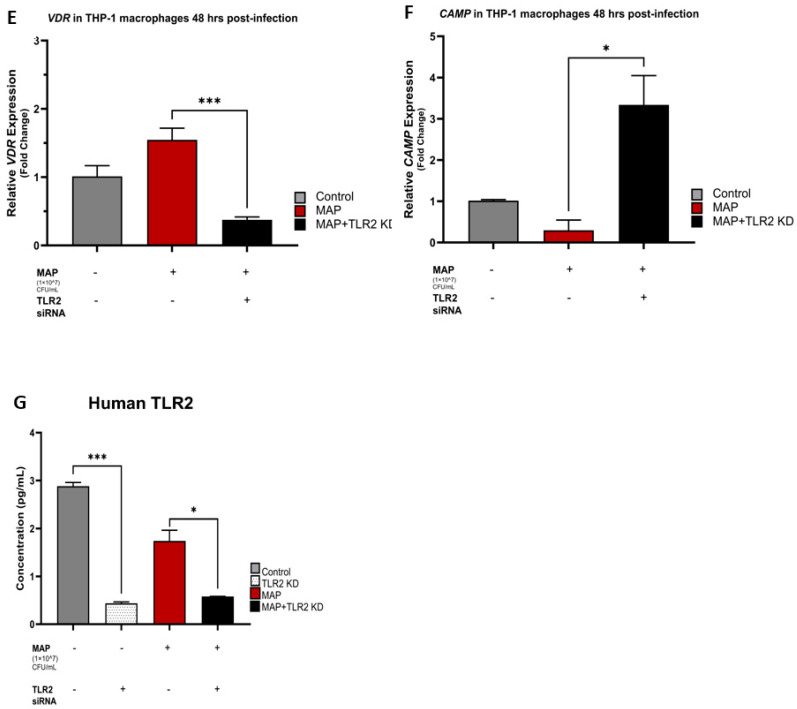
The role of TLR2 in vitamin D activation and Cathelicidin production. Relative expression levels of *CYP27B1* in THP-1 macrophages 24 h (**A**) and 48 h (**D**) post-MAP infection. Relative expression levels of *VDR* in THP-1 macrophages 24 h (**B**) and 48 h (**E**) post-MAP infection. Relative expression levels of *CAMP* in THP-1 macrophages 24 h (**C**) and 48 h (**F**) post-MAP infection. Successful human TLR2 gene knockdown using human TLR2 siRNA was validated with human TLR2 ELISA (**G**). Relative expressions were quantified using RT-qPCR. Statistical analysis was performed on the data obtained from three biological (*n* = 3) and three technical (*n* = 3) replicates. * Indicates *p*-value of less than 0.05. ** Indicates *p*-value of less than 0.01. *** Indicates *p*-value of less than 0.001.

At 24 h post-infection, there was a powerful upstroke in the relative expression of *CYP27B1* and *CAMP* in MAP-infected THP-1 macrophages following TLR2 siRNA transfection with an average increase of 5.55 ± 0.82 and 24.47 ± 6.41 folds, respectively (*p*-value < 0.01) (Figure 2A,C). This was also evident 48 h post-infection with a mean increase in the relative expression fold change of 10.02 ± 0.95 (*p*-value < 0.001) for *CYP27B1* (Figure 2D), and 3.37 ± 0.71 (*p*-value < 0.05) for *CAMP* (Figure 2F) compared to the control, and the MAP-infected only groups. *VDR* relative expression did not show similar associations with TLR2 presence and MAP effects seemed independent of TLR2 siRNA transfection (Figure 2B,E). Successful knockdown of the human TLR2 gene in THP-1 macrophages was validated with human TLR2 ELISA, which showed a statistically significant reduction in human TLR2 concentrations in the siRNA transfected group in comparison to the control non-transfected groups (Figure 2G).

### 4.3. MAP Infection Decreases Total and Phosphorylated p38/MAPK Levels and the Pathway Inhibition Is Independent of TLR2 Presence

**Figure 3:**
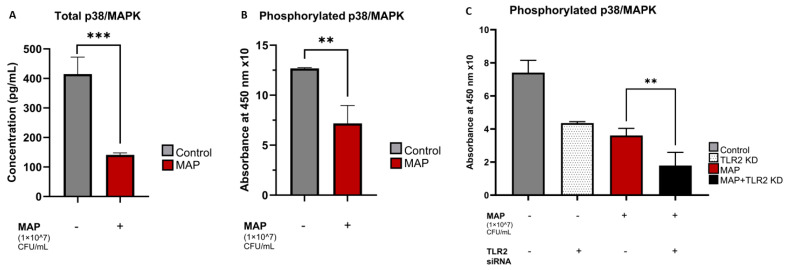
The effects of MAP infection and TLR-2 presence on total and phosphorylated p38/MAPK levels. Total (**A**) and phosphorylated (**B**,**C**) p38/MAPK levels were measured with ELISA. Absorbances at 450 nm reflecting the concentrations of phosphorylated p38/MAPK are multiplied by a factor of 10 for simplicity and displayed on the *y*-axis in (**B**,**C**). Statistical analysis was performed on the data obtained from three biological (*n* = 3) and three technical (*n* = 3) replicates. ** Indicates *p*-value of less than 0.01. *** Indicates *p*-value of less than 0.001.

MAP infection significantly reduced the total levels of p38/MAPK in THP-1 macrophages with a mean concentration of 141.1 ± 6.55 pg/mL in MAP-infected cells compared to 414.7 ± 57.50 pg/mL in the non-infected control group (*p*-value < 0.001) (Figure 3A). MAP-infected groups also showed significantly lower absorbance readings for phosphorylated p38 at 450 nm compared to the control, which indicates decreased levels of the phosphorylated (active) p38 constituents inside MAP-positive macrophages (*p*-value < 0.01) (Figure 3B). MAP infection was still able to cause these effects upon knocking down the TLR2 gene, with the mean absorbance readings for phosphorylated p38/MAPK at 450 nm (multiplied by 10 for simplification) being 7.41 ± 0.74 in the control group, 4.36 ± 0.08 in the TLR2 knockdown group, 3.61 ± 0.43 in the MAP-infected group, and 1.79 ± 0.78 in the MAP-infected, siRNA transfected groups (Figure 3C).

### 4.4. MAP Infection Alters CYP27B1, VDR, and CAMP Expression through Interaction with the p38/MAPK Pathway

**Figure 4:**
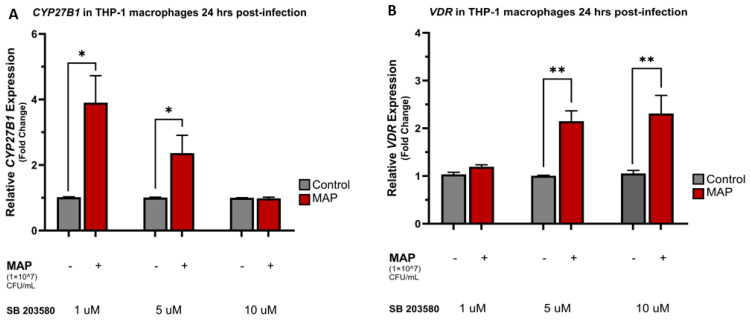

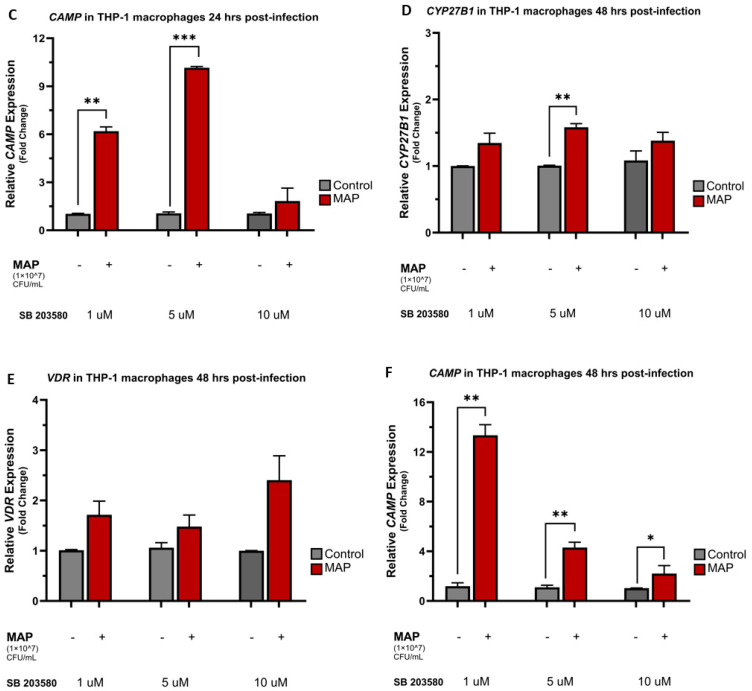
MAP infection effects on *CYP27B1*, *VDR*, and *CAMP* expression through the interaction with the p38/MAPK pathway. Relative expression levels of *CYP27B1* in THP-1 macrophages 24 h (**A**) and 48 h (**D**) post-MAP infection. Relative expression levels of *VDR* in THP-1 macrophages 24 h (**B**) and 48 h (**E**) post-MAP infection. Relative expression levels of *CAMP* in THP-1 macrophages 24 h (**C**) and 48 h (**F**) post-MAP infection. Relative expressions were quantified using RT-qPCR. Statistical analysis was performed on the data obtained from three biological (*n* = 3) and three technical (*n* = 3) replicates. * Indicates *p*-value of less than 0.05. ** Indicates *p*-value of less than 0.01. *** Indicates *p*-value of less than 0.001.

Upon treatment with different concentrations of the p38 antagonist SB 203580, the relative expression of *CYP27B1*, *VDR*, and *CAMP* were all increased compared to the control following 24 h and 48 h of MAP infection. There was an average fold change increase of 3.91 ± 0.82 for *CYP27B1* (*p*-value < 0.05) and 6.19 ± 0.27 for *CAMP* (*p*-value < 0.001) following 24 h of MAP infection and p38 blockade with 1 μM of SB 203580. Similarly, increasing the SB 203580 concentration to 5 μM resulted in the same powerful increase in the expression of *CYP27B1* and *CAMP* with a mean fold change increase of 2.36 ± 0.54 (*p*-value < 0.05) and 10.15 ± 0.09 (*p*-value < 0.001), respectively (Figure 4A,C). MAP infection was noted to exert the same upregulation effects on *CYP27B1* and *CAMP* expression after 48 h of infection and treatment with SB 203580 (Figure 4D,F). p38 antagonism repeatedly led to an increase in the relative expression of *VDR* following 24 and 48 h of MAP infection in comparison to the control non-infected groups (Figure 4B,E).

### 4.5. Calcitriol and p38 Antagonism Reduce Oxidative Stress in Caco-2 Monolayers following Macrophage-Mediated MAP Infection

**Figure 5:**
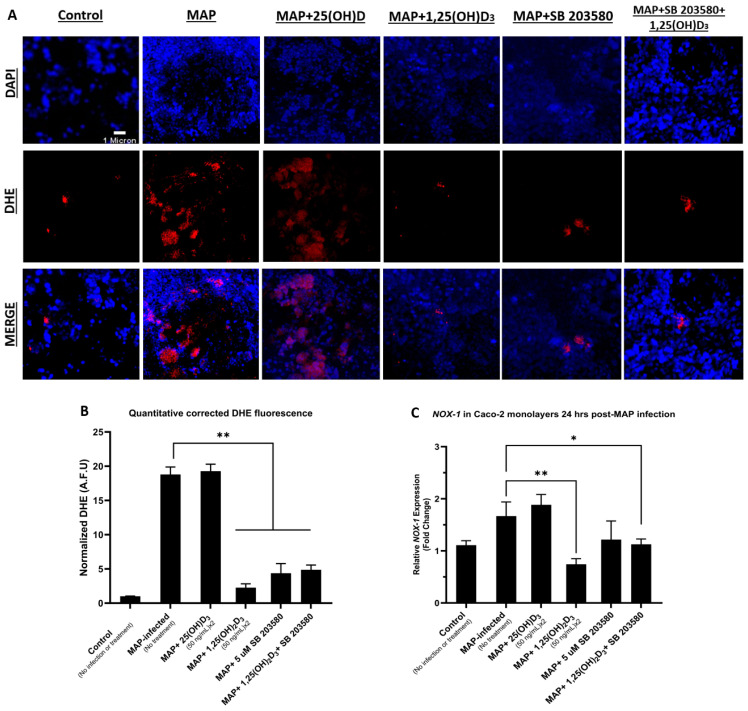
Inhibition of Calcitriol and p38 effects on oxidative stress in Caco-2 monolayers following macrophage-mediated MAP infection. The effects of calcifediol, calcitriol, SB 203580, and a combination of calcitriol and SB 203580 on oxidative stress were measured in Caco-2 monolayers (**A**). Nuclei appear blue and are stained with DAPI. Oxidative stress-positive cells appear red and are stained with DHE, and merged channels for DAPI and DHE appear pink. Quantitative corrected DHE fluorescence integrated density from control and treatment groups (**B**). Relative *NOX-1* expression in Caco-2 monolayers measured 24 h. following THP-1-mediated MAP infection with multiple treatments (**C**). Statistical analysis was performed on the data obtained from three biological (*n* = 3) and three technical (*n* = 3) replicates. * Indicates *p*-value of less than 0.05. ** Indicates *p*-value of less than 0.01.

In Caco-2 monolayers, THP-1 macrophage-mediated MAP infection led to an upregulation of the relative expression of the oxidative stress marker *NOX-1* with a mean fold increase of 1.67 ± 0.27 compared to the control, non-infected groups. MAP infection of the groups treated with calcitriol, SB 208035, and a combination of both were noted to have lower *NOX-1* relative expression compared to the MAP-infected groups only, with an average fold decrease of 0.74 ± 0.11 (*p*-value < 0.01) in the calcitriol-treated group, 1.22 ± 0.36 in the SB 203580 group, and 1.13 ± 0.10 (*p*-value < 0.05) in the calcitriol-SB 203580 combination treatment group (Figure 5C). The results of DHE fluorescence staining confirmed the *NOX-1* expression trends with an elevated arbitrary fluorescence unit (A.F.U) in the MAP-infected groups when normalized to the control, non-infected group. MAP infection following treatments with calcitriol, SB 203580, and a combination of both was noted to have a reduction in DHE fluorescence with normalized A.F.U averages of 2.26 ± 0.57, 4.34 ± 1.41, and 4.88 ± 0.68 (*p*-value < 0.01) in these groups, respectively, compared to 18.79 ± 1.09 in MAP-infected groups without any treatment. Treatment with the inactive form of vitamin D, calcifediol was neither associated with a significant reduction in *NOX-1* expression, nor DHE fluorescence (Figure 5A,B).

### 4.6. Calcitriol and p38 Antagonism Reduce Inflammation Secondary to MAP Infection in THP-1 Macrophages

**Figure 6:**
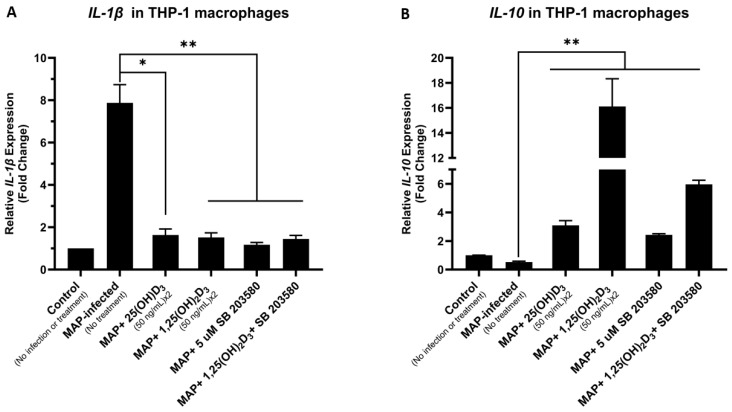
The anti-inflammatory effects of vitamin D isoforms and p38 antagonism on MAP-infected THP-1 macrophages. Relative *IL-1β* expression (**A**) and *IL-10* expression (**B**) in THP-1 macrophages measured 24 h following MAP infection with multiple treatments of calcifediol, calcitriol, SB 203580, and a combination of calcitriol and SB 203580. Statistical analysis was performed on the data obtained from three biological (*n* = 3) and three technical (*n* = 3) replicates. * Indicates *p*-value of less than 0.05. ** Indicates *p*-value of less than 0.01.

MAP-infected THP-1 macrophages had a mean increase of 7.89 ± 0.86 folds in the relative expression of the pro-inflammatory cytokine *IL-1β* compared to the non-infected control group. MAP infections in the groups treated with calcifediol, calcitriol, SB 203580, and a combination of both calcitriol and SB 203580 showed reduced *IL-1β* expression levels of 1.64 ± 0.28 (*p*-value < 0.05), 1.52 ± 0.22, 1.17 ± 0.11, and 1.45 ± 0.17 (*p*-value < 0.01), respectively (Figure 6A). Conversely, the relative expression of the anti-inflammatory cytokine *IL-10* was significantly reduced in the MAP-infected THP-1 macrophages with a mean fold change reduction of 0.52 ± 0.07 in comparison to the control, non-infected groups. Treatments with calcifediol, calcitriol, SB 203580, and a combination of calcitriol and SB 203580 followed by MAP infection were noted to elevate *IL-10* expression levels to an average of 3.09 ± 0.33, 16.12 ± 2.22, 2.43 ± 0.08, and 5.96 ± 0.29 (*p*-value < 0.01) folds in these treatment groups, respectively (Figure 6B).

## 5. Discussion

CD pathogenesis involves an enigmatic interaction of several factors, including genetic susceptibility, immunologic abnormalities, and environmental triggers [23]. One of the most reported microbial etiological agents associated with CD development is MAP, which is also linked to a similar disorder affecting ruminants known as Johne’s disease [24]. The distinctive prevalence of MAP infection in CD results in an inconsistency between the anti-inflammatory and the pro-inflammatory cytokines, leading to a chronic inflammatory response [25]. Previously, we found that a dysregulated pro-inflammatory cytokine level caused by MAP infection results in an extraintestinal manifestation characterized by hyperactive osteoclast activity, which eventually affects bone biomarker levels such as osteocalcin and calcium among CD patients [26]. Additionally, the prevalence of osteoporosis is higher among inflammatory bowel disease patients in general, and some relatively young patients have been diagnosed with severe cases of bone disorders [27].

There is a strong association between vitamin D levels and several disorders with an underlying autoimmune etiology such as rheumatoid arthritis, sarcoidosis, Hashimoto thyroiditis, and type 1 diabetes mellitus [28,29,30,31]. In CD, vitamin D deficiency is not only a by-product of the malabsorptive derangements that occur along the disease course but rather a key player in the pathogenesis of the disorder [32]. Calcitriol is known to have hormonal functions as it controls several transcriptional factors and innate immune responses [32]. Furthermore, genomic-wide association studies (GWASs) have found a link between CD and the expression of nucleotide oligomerization domain 2 (NOD-2), a key player in the innate immune response to bacterial infection and mucosal inflammation [33]. This is necessary to state for the scope of our study for two reasons; firstly, due to the vital role NOD-2 plays in the context of MAP-seropositive CD, and secondly, the fact that NOD-2 expression is influenced by circulating vitamin D inputs [32,33,34,35].

Our recent study showed that the anti-inflammatory role of 1,25(OH)2D3 is mediated by cathelicidin, a peptide with bactericidal and anti-inflammatory properties [14]. We found that serum samples of MAP-positive CD patients had significantly lower calcitriol levels compared to the MAP-seronegative samples. Similarly, another study found that Mtb also modulates cathelicidin synthesis and alters vitamin D activation through interaction with the TLR2 and influencing *CYP27B1* and *VDR* expression [21]. Therefore, we were intrigued to know if MAP utilizes a similar mechanism to ultimately reduce the conversion of inactive vitamin D to its active form.

In this study, we found that MAP infection indeed alters *CYP27B1*, *VDR*, and *CAMP* expression levels in macrophages. Similar to Padhi et al.’s findings with Mtb, there was a downregulation of the expression of both *CYP27B1* and *VDR* following 24 h of MAP infection. In addition, *CAMP* expression was induced by MAP at the same time point. This is in agreement with our hypothesis that MAP infection aims to lower circulating levels of active vitamin D by inhibiting the gene expression of the conversion enzyme that yields calcitriol, as well as the receptor that binds calcitriol and conveys its signals downstream. In addition, *CAMP* expression was ramped up 24 h post-MAP infection, which is expected as the cells try to respond to acute infectious triggers. Moreover, we were curious to know if supplementing calcifediol, as the inactive substrate of *CYP27B1*, would make a difference in these expression trends, and it did. It was found that MAP effects on the expression of all three genes were reversed in THP-1 macrophages when supplemented with calcifediol. We also found that these effects were time-dependent, as the exact opposite was observed 48 h post-MAP infection. This is an indication that time-sensitive defensive cellular responses would take place after the macrophages have been subjected to MAP stressors for 48 h.

We also demonstrated that TLR2 was essential for MAP infection to hinder vitamin D activation and cathelicidin production as we showed that knocking down TLR2 with siRNA resulted in a significantly upregulated *CYP27B1* and *CAMP* expression. The gastrointestinal mucosa is the surface where the luminal contents and submucosal structures interact [36]. Several factors can prevent epithelial injury, such as pH regulation, mucus secretion, and blood flow [36]. In our intestinal epithelial cell culture model (Caco-2 monolayers), THP-1 macrophage-mediated MAP infection led to an upregulation of the relative expression of the oxidative stress marker *NOX-1*, which was confirmed by quantifying the intensity level of the DHE stain. The importance of the nitrite anion in human biology was addressed in two studies [37,38]. Therefore, using an antioxidant such as N-acetylcysteine (NAC) could have substantial protective effects against colonic injury, by regulating free radical production and reducing inflammation [39]. Along the same lines, our data showed that using calcitriol, SB 208035, and a combination of both lowered *NOX-1* relative expression compared to the MAP-infected control. Finally, calcitriol and p38 antagonism reduced the expression of the pro-inflammatory cytokine *IL-1β* and had a positive effect on the anti-inflammatory cytokine *IL-10* in MAP-infected THP-1 macrophages.

## 6. Conclusions

**Figure 7:**
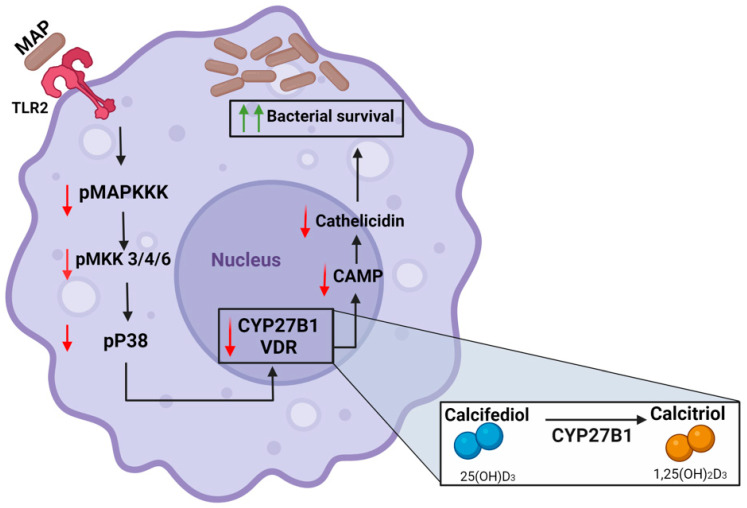
MAP infection mediates vitamin D deficiency in CD patients through inhibition of the TLR2-dependent p38/MAPK-CYP27B1-VDR-CAMP axis. Schematic created with BioRender.com, accessed on 1 February 2024.

Our study indicates that MAP interferes with the activation of vitamin D in infected macrophages. Consequently, an essential step for cathelicidin expression is impeded, which enhances bacterial survival and inflammation. Additionally, our data show that MAP influences the expression of the key vitamin D metabolic enzymes *CYP27B1*, *VDR*, and *CAMP* via interacting with the TLR2-dependent p38/MAPK signaling pathway (Figure 7). Finally, we demonstrated that calcitriol treatment and p38/MAPK blockade reduce cellular oxidative stress and inflammatory markers in intestinal epithelial cells mediated with MAP-infected macrophages. Ultimately, we characterized a novel mechanism by which MAP infection can exacerbate vitamin D deficiency in CD patients, which can explain the resistance to vitamin D supplementation seen in MAP-seropositive CD patients.

## Data Availability

The original contributions presented in the study are included in the article. Further inquiries can be directed to SN.
